# Supplementary material for: Adolescents’ screen time displaces multiple sleep pathways and elevates depressive symptoms over twelve months
Source: PLOS Glob Public Health. 2025 Apr 2;5(4):e0004262. doi: 10.1371/journal.pgph.0004262 (PMC11964217; doi:10.1371/journal.pgph.0004262)
Supplement: S1 Table — Preregistered case extraction procedure. (PDF) [file pgph.0004262.s001.pdf]

S1 Table. Sample selection. Preregistered case extraction procedure.

**Participant Inclusion** [(a)]	**Participant Exclusion**
N = **10 229** (100%) participants in total: Counting both intervention and control group participants	**5192** cases (50.4% of *N*) excluded due to: Primary Intervention Exposure
***n* = 5107** cases (49.6% of *N*): Unexposed to primary intervention.	**236** cases (2.3% of *N*) excluded due to: Suicide Risk Intervention
***n* = 4871** cases (47.3% of *N*): Unexposed to both interventions	**38** cases (0.8% of *N*) excluded due to: Missing or 'Other' Gender
***n* = 4833** (46.9% of *N*): Unexposed 'Boys' and 'Girls'.	**23** cases (0.5% of *N*) excluded due to: Missing or out-of-range Age
***n* = 4810** (46.7% of *N*): Unexposed "Boys" and "Girls", aged 12–16 years	**4810** cases retained through: Multiple imputation of missing values [(b)]
Final sample: N = **4810**; *Median* age = **14 years**; *n* = 2446 boys (**50.9%**) + *n* = 2364 girls (49.1%).	

[(a)] The table reads left to right, from top to bottom. The same sample information has been preregistered.

[(b)] The number of mice datasets (*m* = 70) reflects the proportion of cases with at least one missing datapoint at any wave; including, e.g., missing screen time data at follow-up waves, which were never intended for SEM modelling purposes, but is informative to the mice algorithm.
