# Supplementary material for: Adolescents’ screen time displaces multiple sleep pathways and elevates depressive symptoms over twelve months
Source: PLOS Glob Public Health. 2025 Apr 2;5(4):e0004262. doi: 10.1371/journal.pgph.0004262 (PMC11964217; doi:10.1371/journal.pgph.0004262)
Supplement: S2 Table — Preregistered, Primary, and Secondary SEM fit. (PDF) [file pgph.0004262.s002.pdf]

S2 Table. Model fit indices. Preregistered, Primary, and Secondary SEM fit.

**Model characteristics**	**Dataset (Mice vs. CC)**	**Absolute fit** χ^2; **df; p; SCF**	**Local fit RMSEA (*p*); SRMR**	**Global fit CFI; TLI**
**Preregistered SEM**<br><br>Parsimonious model with all four mediators but no covariances	**Mice:** Multiple imputation data (*N* = 4810 × *m*70)	χ^2 = 5757.85<br>**df** = 754<br>***p*** < 0.001	**RMSEA** = 0.047<br>RMSEA (***p***) = 1.0<br>**SRMR** = 0.060	**CFI** = 0.456<br>**TLI** = 0.378
**Primary SEM**<br><br>Used Chronotype and excluded Social Jetlag as mediator<br><br>Used two mediator covariances:<br>*"Cov.1" and "Cov.2"*	**Mice:** Multiple imputation data (*N* = 4810 × *m*70)	χ^2 =1174.15<br>**df** = 754<br>***p*** < 0.001	**RMSEA** = 0.015<br>RMSEA (***p***) = 1.0<br>**SRMR** = 0.030	**CFI** = 0.945<br>**TLI** = 0.936
	**CC:** Complete case analysis (*N* = 1905)	χ^2 =1482.89<br>**df** = 754<br>***p*** < 0.001<br>**SCF** = 1.57	**RMSEA** = 0.040<br>RMSEA (***p***) = 1.0<br>**SRMR** = 0.045	**CFI** = 0.944<br>**TLI =** 0.935
**Secondary SEM**<br><br>Used Social Jetlag and excluded Chronotype as mediator<br><br>Used two mediator covariances:<br>*"Cov.1" and "Cov.3"*	**Mice:** Multiple imputation data (*N* = 4810 × *m*70)	χ^2 = 1146.49<br>**df** = 754<br>***p*** < 0.001	**RMSEA** = 0.009<br>RMSEA (***p***) = 1.0<br>**SRMR** = 0.037	**CFI** = 0.947<br>**TLI** = 0.939
	**CC:** Complete case analysis (*N* = 1905)	χ^2 = 1444.40<br>**df** = 754<br>***p*** < 0.001<br>**SCF** = 1.57	**RMSEA** = 0.039<br>RMSEA (***p***) = 1.0<br>**SRMR** = 0.042	**CFI** = 0.945<br>**TLI** = 0.937

Abbreviations

CC = Complete Case Analysis (i.e., no multiple imputation data analyzed)

SCF = Satorra–Bentler *Scaling Correction Factor* (only relevant for complete case [CC] analyses)

MICE; Mice; *m* = Multiple Imputation through Chained Equations

Chi-square (χ^2) = Test of absolute model fit: χ^2–test statistic, degrees of freedom (*df*), and probability (*p*) values

RMSEA = Root Mean Square Error of Approximation

SRMR = Standardized Root Mean Squared Residual

CFI = Comparative Fit Index

TLI = Tucker-Lewis Index

Converging models: All presented models converged but the preregistered model had unacceptably poor global fit. This global fit was improved by adding two covariances two each model. *Cov.1*, *Cov.2*, and *Cov.3* model modifiers decrease the models' degrees of freedom but generated acceptable model fit due to the large covariance between sleep mediators. Expressed in lavaan code, the SEM defined the sleep covariance as [WASD~~SQI+CT], or secondarily as [WASD~~SQI+SJL]. The covariance between Chronotype and Social Jetlag was large (*Beta* ≥ 0.85; *p* < 0.001). These modifications made all SEM models converge, but negative variances were detected in 1 of 70 mice datasets (Heywood case warning in *m* = 28). However, as the negative variances could never be found after robust pooling, this dataset was retained and not considered problematic. The imputed data have previously been described in the report "Data Appendix.pdf" accessible from the OSF preregistration site. This appendix also describes how we applied univariate trimming of *Bedtimes* to remove extreme outliers.
