# Supplementary material for: Adolescents’ screen time displaces multiple sleep pathways and elevates depressive symptoms over twelve months
Source: PLOS Glob Public Health. 2025 Apr 2;5(4):e0004262. doi: 10.1371/journal.pgph.0004262 (PMC11964217; doi:10.1371/journal.pgph.0004262)
Supplement: S3 Table — Favoring multigroup setting. (PDF) [file pgph.0004262.s003.pdf]

S3 Table. SEM Information Criteria. Favoring multigroup setting.

Only complete case data	Only complete case data generated all relevant IC	Akaike's IC (AIC)	Bayesian IC (BIC)	Sample size adjusted BIC (SABIC)
(A) The IC from the hypothesized gender-separated SEM	**Multi-group model** (*Boys, n* =904; *Girls*, *n* =1001)	101955.1	103265.4	102515.6
(B) The IC from the same SEM while avoiding gender-dependent model constraints	**Single group model** (*N* =1905)	105584.8	106073.4	105793.8

All information criteria (IC) are interpreted as "smaller is better", thus, the multi-group model is better than the single-group model.
