# Supplementary material for: Adolescents’ screen time displaces multiple sleep pathways and elevates depressive symptoms over twelve months
Source: PLOS Glob Public Health. 2025 Apr 2;5(4):e0004262. doi: 10.1371/journal.pgph.0004262 (PMC11964217; doi:10.1371/journal.pgph.0004262)
Supplement: S4 Table — Unstandardized b-values and their 95% confidence intervals from which the percentage mediation (PM) is calculated. The standardized Beta weights and PM-values are the same as in main manuscript Fig 3. (PDF) [file pgph.0004262.s004.pdf]

S4 Table. Estimated effect sizes for Boys. Unstandardized *b*-values and their 95% confidence intervals from which the percentage mediation (PM) is calculated. The standardized *Beta* weights and PM-values are those displayed in Fig 3.

**Boys'** Effects (paths)	***b*-value** (unstandardized regression coefficient with 95% confidence interval) *Beta*-value (***p*-value**) shown in **Fig 3.**	**Percentage Mediation** (effect ratio) PM = *Numerator / Denominator*
**Sleep Quality Index** Indirect effect (A1×B1) *Numerator*	*b* = **0.003** (95% CI: -0.002 to 0.009)<br>A-*Beta* = 0.052; B-*Beta* = 0.085;<br>AB-*Beta* = 0.004 (*p* = 0.201)	***Boy's SQI mediation***<br>*b* = 0.003 / 0.020 = 0.150.<br>*No mediation* (*p* = 0.201).<br>Only total effect is significant (*p* = 0.015*)
Sleep Quality Index Total effect (A1×B1+C1) *Denominator*	*b* = **0.020** (95% CI: 0.004 to 0.035).<br>C-*Beta* = 0.021<br>ABC-*Beta* = 0.025 (p = **0.015***)	
**Duration (WASD)** Indirect effect (A2×B2) *Numerator*	*b* = **0.003** (95% CI: -0.001 to 0.007)<br>A-*Beta* = -0.202; B-*Beta* = -0.017<br>AB-*Beta* = 0.004 (*p* = 0.152)	***Boy's WASD mediation***<br>*b* = 0.003 / 0.019 = 0.158.<br>*No mediation* (*p* = 0.152).<br>Only total effect is significant (*p* = 0.014*)
Total effect (A2×B2+C2) *Denominator*	*b* = **0.019** (95% CI: 0.004 to 0.034)<br>C-*Beta* = 0.021<br>ABC-*Beta* = 0.024 (*p* = **0.014***)	
**Chronotype** Indirect effect (A3×B3) *Numerator*	*b* = **0.003** (95% CI: -0.001 to 0.007)<br>A-*Beta* = 0.261; B-*Beta* = 0.010<br>AB-*Beta* = 0.003 (*p* = 0.143)	***Boy's Chronotype mediation***<br>*b* = 0.003 / 0.019 = 0.158.<br>*No mediation* (*p* = 0.143).<br>Only total effect is significant (*p* = 0.014*)
Total effect (A3×B3+C3) *Denominator*	*b* = **0.019** (95% CI: 0.004 to 0.034)<br>C-*Beta* = 0.021<br>ABC-*Beta* = 0.024 (*p* = **0.014***)	
**Social Jetlag** Indirect effect (A4×B4) *Numerator*	*b* = **0.002** (95% CI: -0.001 to 0.004)<br>A-*Beta* = 0.189; B-*Beta* = 0.011;<br>AB-*Beta* = 0.024 (*p* = **0.017***)	***Boy's Social Jetlag mediation***<br>*b* = 0.002 / 0.019 = 0.105.<br>*No mediation* (*p* = 0.190).<br>Only total effect is significant (*p* = 0.017*)
Total effect (A4×B4+C4) *Denominator*	*b* = **0.019** (95% CI: 0.003 to 0.034)<br>C-*Beta* = 0.018<br>ABC-*Beta* = 0.002 (*p* = **0.190**)	

* *p* < 0.05

PM = The effect ratio (Percentage Mediation, PM) was calculated by dividing the indirect effect over the total effect using unstandardized beta weights with three decimals. Standardized Beta weights ("Std.all") are displayed alongside each *b*-value to increase their comparability and interpretability.
