# Supplementary material for: Adolescents’ screen time displaces multiple sleep pathways and elevates depressive symptoms over twelve months
Source: PLOS Glob Public Health. 2025 Apr 2;5(4):e0004262. doi: 10.1371/journal.pgph.0004262 (PMC11964217; doi:10.1371/journal.pgph.0004262)
Supplement: S5 Table — Unstandardized b-values and their 95% confidence intervals from which the percentage mediation (PM) is calculated. The standardized Beta weights and PM-values are the same as in main manuscript Fig 3. (PDF) [file pgph.0004262.s005.pdf]

S5 Table. Estimated effect sizes for Girls. Unstandardized *b*-values and their 95% confidence intervals from which the percentage mediation (PM) is calculated from. The standardized *Beta* weights and PM-values are those displayed in Fig 3.

**Girls'**<br>Effects (paths)	***b*-value** (unstandardized regression coefficient with 95% confidence interval)<br>*Beta* weight (***p*-value**) shown in **Fig 3.**	**Percentage Mediation**<br>(effect ratio) PM = *Numerator* / *Denominator*
**Sleep Quality Index**<br>Indirect effect<br>(A1×B1)<br>*Numerator*	*b* = **0.017** (95% CI: 0.005 to 0.029)<br>A-*Beta* = 0.141; B-*Beta* = 0.142<br>AB-*Beta* = 0.020 (*p* = **0.004***)	***Girl's SQI mediation***<br>*b* = 0.017 / 0.030 = 0.57<br>Significant SQI mediation<br>**PM = 57%** (*p* = 0.004*)<br>Direct effect is also significant<br>(*p* = 0.025)
Sleep Quality Index<br>Total effect<br>(A1×B1+C1)<br>*Denominator*	*b* = **0.030** (95% CI: 0.003 to 0.057)<br>C-*Beta* = 0.015<br>ABC-*Beta* = 0.035 (*p* = **0.028***)	
**Duration (WASD)**<br>Indirect effect<br>(A2×B2)<br>*Numerator*	*b* = **0.008** (95% CI: 0.001 to 0.016)<br>A-*Beta* = -0.234; B-*Beta* = -0.042;<br>B-*Beta* = 0.010 (*p* = **0.024***)	***Girl's WASD mediation***<br>*b* = 0.008 / 0.021 = 0.38<br>*Significant WASD mediation*<br>**PM = 38%** (*p* = 0.024*)<br>No direct effect remaining<br>(*p* = 0.115) **
Total effect<br>(A2×B2+C2)<br>*Denominator*	*b* = **0.021** (95% CI: -0.005 to 0.048)<br>C-*Beta* = 0.015<br>ABC-*Beta* = 0.025 (*p* = **0.115**)	
**Chronotype**<br>Indirect effect<br>(A3×B3)<br>*Numerator*	*b* = **0.010** (95% CI: 0.000 to 0.019)<br>A-*Beta* = -0.304; B-*Beta* = 0.037<br>AB-*Beta* = 0.011 (*p* = **0.040***)	***Girl's Chronotype mediation***<br>*b* = 0.010 / 0.022 = 0.45<br>Significant *chronotype mediation*<br>**PM = 45%** (*p* = 0.040*)<br>No direct effect remaining<br>(*p* = 0.074) **
Total effect<br>(A3×B3+C3)<br>*Denominator*	*b* = 0.022 (95% CI: -0.002 to 0.047)<br>C-*Beta* = 0.015<br>ABC-*Beta* = 0.026 (*p* = 0.074)	
**Social Jetlag**<br>Indirect effect<br>(A4×B4)<br>*Numerator*	*b* = **0.005** (95% CI: -0.001 to 0.011)<br>*A-Beta* = 0.227; B-*Beta* = 0.026<br>AB-Beta = 0.006 (*p* = **0.094**)	***Girl's Social Jetlag mediation***<br>*b* = 0.005 / 0.020 = 0.250<br>*No mediation* (*p* = 0.094)<br>Total effect is not significant<br>(*p* = 0.105)
Total effect<br>(A4×B4+C4)<br>*Denominator*	*b* = **0.020** (95% CI: -0.004 to 0.045)<br>C-Beta = 0.018<br>ABC-*Beta* = 0.024 (*p* = **0.105**)	

* *p* < 0.05

** "*Full mediation*" is indicated – but is a term designating the test of a null hypothesis (that the C-path is non-significant after AB-adjustment) which was not formally tested in this study. The expression "*Full mediation"* is just a commonly used label for mediation results wherein the indirect effect is significant while the direct effect is not.
