# Supplementary material for: Adolescents’ screen time displaces multiple sleep pathways and elevates depressive symptoms over twelve months
Source: PLOS Glob Public Health. 2025 Apr 2;5(4):e0004262. doi: 10.1371/journal.pgph.0004262 (PMC11964217; doi:10.1371/journal.pgph.0004262)
Supplement: S6 Table — Scores compared to excluded group. (PDF) [file pgph.0004262.s006.pdf]

S6 Table

S6 Table. Screen time estimate bias check. Scores compared to excluded group.

Sample pool group	N	M	SD	Median (interquartile range; IQR)	Group difference (t-test)
Group 1: Excluded or ineligible cases with valid data	5189	3.16	1.04	*Md* = 3 (IQR = 2.4 to 3.8)	*Both groups merged* (*N = 9780*)*: M = 3.12* (SD = 1.01)<br><br>*Group Mean Difference* = 0.09 (95% CI: 0.05 to 0.13) $t_{(df = 9778)}$ = 4.29; p < 0.001* Cohen's d = 0.09
Group 2: Included, eligible cases with valid data	4591	3.07	0.97	*Md* = 3 (IQR = 2.3 to 3.6)	

A non-preregistered independent samples t-test was conducted, with Listwise deletion applied to complete case data. The purpose was to examine whether the observed screen time scores in the extracted sample were equal to the mean among excluded cases (this group is defined in S1 Table in S1 Supplementary Information). The calculated average screen time scores were based on wave T1 measurement only, using the first screen time item and the two leisure online hour items (the two latter assumed a weekly cycle of five schooldays and two weekend days).

* Note that this t-test was quite over-powered to detect small group differences, reflected by the small Cohen's d value (d = 0.09).
