# Supplementary material for: Adolescents’ screen time displaces multiple sleep pathways and elevates depressive symptoms over twelve months
Source: PLOS Glob Public Health. 2025 Apr 2;5(4):e0004262. doi: 10.1371/journal.pgph.0004262 (PMC11964217; doi:10.1371/journal.pgph.0004262)
Supplement: S7 Table — Detailed results. (PDF) [file pgph.0004262.s007.pdf]

S7 Table

S7 Table. Invariance testing. Detailed results.

**Measurement and Conclusion**	**Weak/metric invariance**	**Strong/scalar invariance**
**Screen time:** Observed weak gender invariance in the screen time metric	Weak/metric invariance was found. Partial weak/metric invariance could not be assessed because two items were in need to be freely estimated; and as the scale only consisted of three items, this made the configural and invariance model have equal degrees of freedom.	Partial strong/scalar was found with one factor loading and one intercept (screen time item 1 [B53]) freely estimated ($F_{(2,3096.3)} = 2.110$; $p = 0.122$).
**Sleep Quality Index (SQI):** Observed partial gender invariances in the SQI metric	Partial weak/metric was found with one ('4: *Disturbed sleep*' [M47_7]) freely estimated factor loading ($F_{(2,541.74)} = 1.446$; $p = 0.236$).	Partial strong/scalar invariance was found with one loading ('4: *Disturbed sleep*' [M47_7]) and two intercepts ('2: *Repeated awakenings*' [M47_3]; '4: *Disturbed sleep'* [M47_7]) freely estimated ($F_{(3,547.38)} = 1.389$, $p = 0.245$).
**General depression:** Observed partial gender invariance in the G-factor metric	Partial weak/metric invariance was found with five (BDI-II items: 2, 10, 13, 14, 12; [Y25_B, _J, _M, _N, _L]) factor loadings freely estimated ($F_{(34,80.919)} = 0.203$; $p = 1.0$).	Partial strong/scalar invariance was found with five loadings (BDI-II items: 2, 10, 13, 14, and 12; [Y25_B, _J, _M, _N, _L]) and four intercepts (BDI-II items: 1, 4, 6, and 10; [Y25_A, _D, _F, _J]) freely estimated ($F_{(48,88.901)} = 0.410$; $p = 1.0$).

Summary interpretation: The results yielded only partial metric and scalar invariance combinations for the constructs. This indicates that the interpretation of the constructs differs between the genders and that divergent path coefficients can be due to these differences, or to communalities in parts of the constructs (i.e. partial invariance). The result may be interpreted to indicate that these three latent constructs differ between boys and girls such that structural paths can be due to either real differences in common parts of the latent constructs or latent constructs parts that differ between the genders.
