# Supplementary material for: Adolescents’ screen time displaces multiple sleep pathways and elevates depressive symptoms over twelve months
Source: PLOS Glob Public Health. 2025 Apr 2;5(4):e0004262. doi: 10.1371/journal.pgph.0004262 (PMC11964217; doi:10.1371/journal.pgph.0004262)
Supplement: Attachment — Submitted filename: PGPH_Response to auditor_PGPH-D-24-02411.docx [file pgph.0004262.s008.docx]

Dear Risalyn Banlaygas, audits and editors of PLOS Global Public Health,

Regarding manuscript: PGPH-D-24-02441, entitled “Adolescents’ screen time displaces multiple sleep pathways and elevates depressive symptoms over twelve months”.

Thank you for reminding us about these journal requirements. We hope that we have addressed all relevant issues.

New files have been uploaded:

As requested, we have uploaded additional documents to the PLOS Editorial Manager (EM). These documents were gathered in a compressed zip-folder, named “Ethical and Preregistration documents_PGPH-D-24-02441”. It has been uploaded as an “Other” file. It contains five PDF documents: (1) The form you sent us to fill in, i.e., the “Human Participants Research Checklist”; and the Ethical Approval in (2) Swedish original; and (3) An English translation of it. The zip-folder also contains (4) A copy of the preregistration; and (5) Preregistration data appendix.

Figures have been PACE-checked:

We have re-rendered Fig 1-3.tif and checked their quality by using the recommended PACE tool. We hope this improved the figures.

Minor formatting changes of the manuscript:

We have made some slight formatting corrections to the manuscript, to adhere to the author formatting guidelines you pointed our attention towards. We have uploaded the revised manuscript with tracked changes, as well as a “clean” version. We have also deleted the former, obsolete, files. We have changed the following as per your request: (1) The method section of the manuscript now includes exact dates of data collection and when data became accessible to authors (row: 217-219 in the “clean” document). Please note that the same information is found in Section 7 in the Preregistration document (i.e., document 4 mentioned above). We have also (2) changed the captions of the supplementary tables (from e.g. “Appendix S1, Table S1”, to “S1 Table”). Likewise, we have updated the table names in the supporting information file (S1-S7 Tables); but without tracked changes.

We hope we have addressed all your current concerns. Please let us know whether any further changes or additional information is required, or if we have misinterpreted your request. Again, we thank you for this reminder and look forward to your response.

Best regards

Sebastian Hökby, on behalf of all authors
