# Supplementary material for: Adolescents’ screen time displaces multiple sleep pathways and elevates depressive symptoms over twelve months
Source: PLOS Glob Public Health. 2025 Apr 2;5(4):e0004262. doi: 10.1371/journal.pgph.0004262 (PMC11964217; doi:10.1371/journal.pgph.0004262)
Supplement: Attachment — Submitted filename: Response to reviewers_PGPH-D-24-02441.docx [file pgph.0004262.s009.docx]

# Response to Reviewer #1: Dr. Kjell Ivar Øvergård.

## General comment from reviewer.

Dear Authors,

I have enjoyed reading the manuscript and I have only a few minor comments and requests for amendments. When my comments or requests for amendments I think this paper can be ready for publication.

General response from authors.

Dear reviewer,

As authors, we are very thankful for your scrutinizing review of our paper, your overall assessment of its quality, and your valuable suggestions to improve it. We hope we have interpreted them correctly, and that we have addressed all current concerns in the revised manuscript. Some revisions are influenced by the other reviewer and the journal/editor. We address your concerns point by point below, explaining each revision.

Besides what is detailed below, we have address issues in the reference list. We updated some references, which also conferred changes to the text. Most changes were applied to section “Introduction: public health policies for screen time”. For example, Australia might soon make it illegal for people aged <16 years to have social media accounts, so previous references can soon be outdated. We therefore provide the reader with easier access to WHO guidelines while also illustrating how ever-changing this research/policy field is.

## Reviewer comment 1.

Sentence 145-146: Have they done any analysis to check the distirbution of confounders in the sample vs. the population?
Author response 1.

No, we have not performed such analyses. Thank you for directing our attention towards this issue. We have added clarifications during revision and revised the section “methodological strengths and limitations”. Please refer to “Introduction” (**Rows: 160-164**) and Effect interpretations (**Rows: 646-650**) in the Revised, Clean Manuscript. In the revised manuscript, we now state that it is possible that our sample is slightly healthier than the population average, as we quite obviously focus on adolescents who are healthy enough to go to school. Risk groups which we have definitely not captured are for instance hospitalized individuals, or those displaying stable patterns of school refusal; the latter which according to the Swedish School Inspection constituted around 0.17% of this population in 2016 (the starting year of the data collection [Accessible from: https://www.skolinspektionen.se/globalassets/02-beslut-rapporter-stat/granskningsrapporter/tkg/2016/langvarig-franvaro/omfattande-ogiltig-franvaro-kvantitativ-rapport.pdf]). Problematic truancy has an estimated prevalence of 1.8% in Sweden and may also be underrepresented by our data. The data collection took place in classrooms, naturally requiring school attendance, and in turn, naturally requiring some degree of wellbeing. We now describe these issues in the revised manuscript.

Confounders which we have mentioned previously, which we suspect could be important, but left unassessed for generalizability, are individual differences in social relations, family relations, and socioeconomic status (SES). Furthermore, it is not farfetched to believe that these confounders vary between schools, or school climates. But as previously hinted, we perceive current evidence as heterogenous regarding the relative importance of such confounders; at least to the extent that we felt unconfident preregistering them as essential control variables. Exceptions which are already accounted for, or recognized as potentially confounding, include: gender, age, education grade, suicidality, and geographical location (municipality, school). We have declared some as problematic (exclusion of non-binary gender) or accounted for them through statistical adjustment (binary gender), or eligibility criteria (age, suicidality) or study design (school/education grade, geographical coverage, cluster-randomization of schools). As stated in the previous manuscript version, socioeconomic status in this participant pool has been examined in two previous studies (Hökby et al 2023; Lemke et al 2023; the latter also registered under the same parent project at www.OSF.io) and does not seem to interact problematically with screen time, sleep, or depression.

## Reviewer comment 2.

In primary model with the path coefficients you have chosen to present only the p-values for non-statistically significant paths. Please replace the p-values with the path coefficients (but show that it iis not significant by hte lack of a *). so we can see the size of the effect irrespective if it is not statistically significant.

Author response 2.

We agree that your suggested way is a better way to present the results and improves readability. We have made these changes to **Fig 3.** and in some other instances of the revised manuscript, in accordance with your sixth remark. Please refer to **Fig 3** and the revisions in section “Results: effect interpretations”: Rows **519-523** and **538-544** in the revised/clean manuscript.

## Reviewer comment 3.

Lines 378-380. Argument is difficult ot follow: How can both two-tailed tests and one-tailed thests inflate type-II error? Compared to each other one of them would inflate the type-II error more than the other? Would not hte difference betweeen one-tailed and two-tailed be that one-tailed used the 95 percentile as a cut-off for «statistical significance» while a two-tailed tests uses the 97.5 percentile as a cut-off? (of course given that the effect is in the expected direction - as you noted in line 382-384.

Author response 3.

We agree that our way of expressing this argument lacks some clarity and think that some of this text could even be removed to improve clarity. We tried to covey the following message: All of our “directed hypotheses” were preregistered as two-tailed tests (which, as you mention, uses the two 97.5 percentiles to indicate significance); and we did so despite the fact that we would not, on a purely theoretical basis, have accepted significance on the opposite direction (the theoretical model would have “fallen apart”, so to speak). We used two-tailed tests to make our tests even more conservative from the beginning (or “fragile” as we wrote in the manuscript). We could very well have used one-tailed tests for this study, which would have been more powerful in obtaining statistically significant results (due to the 95-percentile indicator of significance, which you also mention). However, we believe that more conservative tests are appropriate for our high-powered analyses. Please refer to **Rows: 398-406** in the revised/tracked manuscript. We hope to have clarified the issue.

## Reviewer comment 4.

Line 392-397ff. You write «The results indicated no measurement invariance in screen time, suggesting that the scale would generate a metric value regardless of gender amongst responders». The usage of measurement invariance is used in the opposite way it is meant to. Measurement invariance is found when a scale works the same way for different demographic groups - which is hte opposite way the term is used in the current manuscript. So, in the quited paragraph the scales actually show measurement invariance since the scores are regardless of the respondes gender. It seems to me that the usage of the term invariance varies in the section 392-404. Please ensure that «measurement invariance» is used in the correct way throughout the paper.
Author response 4.

Thank you for spotting this error. They are indeed “double typos” related to the double negation inherent to the term ‘invariance’. We have double-checked the test results, and they are correct, but we felt the need to rewrite this part towards the end of the Method section, to fully clarify them (**Rows: 414-431** in revised/clean manuscript). Please also see revised **S7 Table,** where we have made similar changes.

## Reviewer comment 5.

In table 1: please indicate the direction of the scales - General (G-factor), Cognitive-Affective and Somatic-Vegetative. Please indicate what is best and what is worst.
Author response 5.

Thank you for pointing out that this information was missing. We have added this information to Table 1. The revised manuscript now states: “(Lower is better)”, in **Table 1**.

## Reviewer comment 6.

line 484 and 489 - and in rest of the paper. Please include the path coefficients also for statistical insignificant paths.
Author response 6.

We agree that this will improve the manuscript. Please refer to **Fig 3** and the revisions in section “Results: effect interpretations”: **519-523** and **538-544** in the revised/clean manuscript. Please also note that we have corrected some minor numeric typos (none of which changes any conclusions. To give an example, one typo regards the direct effect for boys, mentioned in the Abstract, which we believe should not be the sleep-unadjusted Beta weight (0.04; p<0.001), but instead the remaining direct effect (Beta = 0.024; p=0.038).

## Reviewer comment 7.

S4Table: Please use same wording for total effects for Social Jetlag as you have for the other time estimates.
Author response 7.

Thank you for your sharp eye! Indeed, this wording was not used consistently in the previous version. We have changed this in the revised **S4 Table** (lower-right cell and its corresponding left-hand cell). It now reads: “Only total effect is significant (*p* = 0.017*)”, instead of “Significant total effect only (*p* = 0.017*)”. Please refer to the revised **S1 Supplementary Information**, which does not contain tracked changes like the manuscript. Some additional typo fixes, formatting changes, and inconsistency corrections, were made to both **S4 Table** (boys) and **S5 Table** (girls), including their footnotes.

## Reviewer comment 8.

S6Table: Please check and correct the 95 % CI for «All cases with valid screen time data» as the 95% CI clearly cannot be [0.05; 0.13] when the mean is 3.12.

Author response 8.

Thank you for this useful comment. We believe this is not actually an error per se, but perhaps a suboptimal way of conveying statistics within **S6 Table**. This was better expressed in rows **337-340** (previous manuscript), where this table was referenced. Please see Rows **336-340** in the Revised/Clean manuscript. We totally understand how one could interpret **S6 Table** numbers (M; 95% CI) the way you describe it, so we have revised the structure of the table. If we have interpreted your comment correctly, the miscommunication is that the reported 95% CI is not a confidence interval around a Mean, but around the (t-tested) group difference in Means (excluded vs. included subjects = “M diff.”). Thus, the essential message we try to convey is simply the following group difference: *Mean* difference = 0.09 (95% 0.05-0.13) [t = 4.29; df=9778; p < 0.001]. We also added the following sentence under **S6 table**: “*Note that this t-test has high power to detect small group differences, reflected by the small Cohen’s d value (d=0.09)*.” Please see our revision of S6 table in the S1 Supplementary Information.

Best of regards,

Sebastian Hökby, on behalf of all authors.

# Response to Reviewer #2: Dr. Jonathan Vicente dos Santos Ferreira.

## Reviewer #2, comment 1/1.

Dear authors, congratulations for this incredible and well done research.
I do not have much recommendations in the manuscript, otherwise, I suggest to take a look into some grammar mistakes which is common when it comes to not English speakers, and I do definitely understand this.

Besides this, congratulations in this incredible work.

## Author response to reviewer #2, comment 1/1.

Dear reviewer,

As authors, we are very thankful for your scrutinizing review of our paper and overall assessment of its quality. Frankly, we were flattered by your words. Also, thank you for pointing out language errors in the manuscript, which we have tried to correct. We have also corrected some numerical typos that we found (minor numerical typos which do not change any conclusions). We have also made changes (e.g. to some references) suggested by the other reviewer and the journal/editor. Again, thank you for this very encouraging review.

Best of regards,

Sebastian Hökby, on behalf of all authors.
